# Equipping the American Joint Committee on Cancer staging for resectable pancreatic ductal adenocarcinoma with tumor grade: a recursive partitioning analysis

**DOI:** 10.1007/s12032-016-0839-4

**Published:** 2016-10-11

**Authors:** Yu-Tong Chen, Ze-Ping Huang, Zhi-Wei Zhou, Ming-Ming He

**Affiliations:** 1 Department of Medical Oncology, Sun Yat-sen University Cancer Center; State Key Laboratory of Oncology in South China, Collaborative Innovation Center for Cancer Medicine, 651 Dong Feng Road East, Guangzhou, 510060 China; 2 Department of General Surgery, Lan Zhou University Second Hospital, Lanzhou, 730030 China; 3 Department of Gastric and Pancreatic Surgery, Sun Yat-sen University Cancer Center; State Key Laboratory of Oncology in South China, Collaborative Innovation Center for Cancer Medicine, Guangzhou, 510060 China

**Keywords:** Pancreatic ductal adenocarcinoma, American Joint Committee on Cancer staging, Recursive partitioning analysis, Surveillance, Epidemiology, and End Results, Extrapancreatic extension

## Abstract

Previous studies of pancreatic ductal adenocarcinoma (PDAC) have demonstrated that the addition of tumor grade to the 7th American Joint Committee on Cancer (AJCC) staging can provide improved prognostication and that the recently proposed 8th edition AJCC staging exhibited superior reproducibility to the 7th edition in resectable PDAC. Thus, we aimed to combine tumor grade and 8th AJCC stage to develop a refined staging scheme for resectable PDAC. We analyzed 7719 patients with resectable PDAC from the 2004–2012 Surveillance, Epidemiology, and End Results database. We performed recursive partitioning analysis (RPA) to objectively incorporate tumor grade with 8th AJCC stage into a novel staging system. The performance of the proposed RPA staging was assessed against the 8th AJCC staging in terms of discriminatory ability and prognostic homogeneity. For each 8th AJCC stage, survival was significantly worse for high-grade versus low-grade tumors. RPA divided resectable PDAC into five stages: RPA-IA (low-grade T1N0), RPA-IB (high-grade T1N0 or low-grade T2N0), RPA-IIA (high-grade T2N0 or low-grade T3N0/T1–T3N1), RPA-IIB (high-grade T3N0/T1–T3N1 or low-grade T1–T3N2), and RPA-III (high-grade T1–T3N2; median survival: 42, 26, 19, 15, and 12 months, respectively; *P* < 0.001). The RPA staging outperformed the 8th AJCC classifications in terms of discrimination (concordance index, 0.585 versus 0.565; *P* < 0.001) and prognostic homogeneity. Tumor grade can provide additional prognostic information to the 8th AJCC staging. The proposed RPA staging is a superior risk-stratified tool to the 8th AJCC staging and is not substantially more complex.

## Introduction

Pancreatic ductal adenocarcinoma (PDAC) is the most common malignancy in the pancreas and the seventh leading cause of cancer-related death worldwide [1]. Radical resection offers the only chance for cure, but patients with resectable PDAC have high incidence of postsurgical recurrence and dismal prognosis [2].

Conventionally, the outcomes of resectable PDAC are predicted based on the American Joint Committee on Cancer (AJCC) TNM classification, which involves the tumor invasion depth and lymph node status [3]. However, these clinicopathological factors cannot present a complete prognostic picture, and survival within a particular stage is highly variable [4].

Several previous studies have demonstrated the prognostic value of tumor grade in patients with PDAC [4–8]. Additionally, Wasif et al. [7] combined tumor grade and the 7th AJCC stage into a new staging scheme which exhibited superior survival discrimination to the 7th AJCC staging scheme. However, this new staging was based on arbitrarily advancing patients in the presence of high tumor grade to the next higher stage level, which is methodologically not sound [9]. Moreover, several studies have questioned the clinical relevance and reproducibility of the 7th AJCC T and N classification for patients with PDAC [10].

In the recently proposed 8th AJCC staging scheme [9], tumor size was the only accounted factor to determine the T classification for resectable PDAC (T1, T2, and T3: ≤2 cm, >2 cm and ≤4 cm, and >4 cm, respectively) regardless of the involvement of peripancreatic soft tissue, whereas node-positive disease was further classified into N1 (1–3 positive nodes) and N2 stage (≥4 positive nodes). In a recent multi-institutional study of patients with resectable PDAC, although the reproducibility of the 8th edition T classification was superior to the 7th edition, the predictive accuracy of the 7th and 8th AJCC staging schemes was comparable, suggesting a room for improvement of the 8th staging [9].

In the present study, we developed a refined staging scheme for resectable PDAC by using the recursive partitioning analysis (RPA) which can achieve the optimized combination of tumor grade and 8th AJCC stage. The aim of this study is to improve the prognostic performance of the 8th AJCC staging without increasing complexity.

## Patients and methods

### Study cohort

The National Cancer Institute’s Surveillance, Epidemiology, and End Results (SEER) program collects cancer incidence, treatment, and survival data from 18 population-based cancer registries covering approximately 28 % of the US population. Using the SEER database (18 registries), we identified 17,379 patients with PDAC (ICD-O-3 codes: 8140, 8150, 8210, 8211, 8251, 8260, 8261, 8263, 8480, 8481, 8490, 8500, and 8503) from January 2004 to December 2012. Patients with a history of prior malignancy, carcinoma in situ, locally unresectable tumor (T4 classification of the 6th edition AJCC scheme, which is identical with the 7th edition), distant metastasis, and missing information regarding tumor grade, tumor size, 6th AJCC M classification, and number of positive lymph nodes were excluded. The final study cohort consisted of 7719 patients.

Examined covariates included race, age, gender, and marital status, year of diagnosis, SEER region, tumor grade, tumor location, tumor size, positive node count, and examined node count. All patients were restaged by the 8th AJCC staging scheme. Tumor grades 1 and 2 were defined as a “low-grade” group and tumor grades 3 and 4 as a “high-grade” group.

### Statistical analysis

Overall survival (OS) was the primary outcome of interest. Multivariate Cox regression was used to examine the association between tumor grade and hazard ratio (HR) for death after adjusted for other clinicopathologic factors. The Kaplan–Meier method and log-rank tests were used to compare OS between patients with low-grade and high-grade tumors within each 8th AJCC stage.

To develop a refined staging system which incorporated tumor grade together with 8th AJCC stage, recursive partitioning analysis (RPA) [11, 12] was performed to derive new RPA stages by objectively regrouping the following ten patient subgroups: low- and high-grade 8th IA (T1N0), low- and high-grade 8th IB (T2N0), low- and high-grade 8th IIA (T3N0), low- and high-grade 8th IIB (T1–T3N1), and low- and high-grade 8th III (T1–T3N2). The RPA algorithm is based on the optimized binary partition of these subgroups which results in new subgroups with relatively homogeneous survival performance [11, 12]. Multivariate Cox regression was used to examine the association between the RPA stage and hazard ratio (HR) for death after adjustment for clinicopathologic factors. Internal validation of the RPA staging scheme was performed by using bootstrap with 1000 resamples, which quantified model overfit.

The prognostic performance of the RPA staging scheme was assessed against the 8th AJCC staging scheme in terms of discrimination and prognostic homogeneity. The discriminatory capacity of the staging schemes was quantified using the concordance index (C-index) [13]. The value of the C-index ranges from 0.5 to 1.0, with 0.5 indicating a random chance and 1.0 indicating a perfect ability to correctly discriminate the outcome with the staging system; that is, the larger C-index, the superior discriminatory capacity. Additionally, we evaluated the prognostic homogeneity of the RPA staging scheme against the 8th AJCC staging scheme: Within each RPA stage, OS by 8th AJCC stages was compared using the Kaplan–Meier method with log-rank tests.

Statistical significance was set as *P* < 0.05 in a two-tailed test. The statistical analyses were performed using SAS v. 9.3 (SAS Institute, Cary, NC, USA), IBM SPSS Statistics for Windows v.19.0 (IBM Corp., Armonk, NY, USA), and R v. 3.3.1 (http://www.r-project.org).

## Results

Table 1 summarizes the patient characteristics of the study cohort (7719 cases). The majority of the patients were classified as 8th AJCC T2 disease (59.2 %) and node-positive disease (65.7 %). The median numbers of positive and examined node counts were 1 (interquartile range [IQR] 0–3) and 13 (IQR 8–20), respectively. The median survival for patients in the study cohort was 18 months.

**Table 1:** Clinicopathologic characteristics of the study cohort of patients with resectable PDAC (*N* = 7791)

Variable	Median (IQR)/*N* (%)
Age, years	66 (58, 74)
*Race*	
White	6332 (82.0 %)
Black	806 (10.4 %)
Other	581 (7.6 %)
*Sex*	
Male	3906 (50.6 %)
Female	3813 (49.4 %)
*Marital status*	
Married	4803 (62.2 %)
Unmarried	203 (2.6 %)
Unknown	2713 (35.1 %)
*Year of diagnosis*	
2004-2006	2177 (28.2 %)
2007-2009	2678 (34.7 %)
2010-2012	2864 (37.1 %)
*SEER region*	
Midwest	1199 (15.5 %)
Northeast	1454 (18.8 %)
South	1382 (17.9 %)
West	3684 (47.7 %)
*Tumor site*	
Head	5993 (77.6 %)
Body	440 (5.7 %)
Tail	602 (7.8 %)
Not specified	684 (8.9 %)
*Tumor grade*	
I/II	4863 (63.0)
III/IV	2856 (37.0)
*Tumor size*	31 (25, 40)
≤2 cm (8th T1)	1333 (17.3 %)
>2 cm and ≤4 cm (8th T2)	4569 (59.2 %)
>4 cm (8th T3)	1817 (23.5 %)
*Positive node count*	1 (0, 3)
0 (8th N0)	2650 (34.3 %)
1–3 (8th N1)	2571 (33.3 %)
≥4 (8th N2)	2498 (32.4 %)
Examined node count	13 (8, 20)

*PDAC* pancreatic ductal adenocarcinoma, *IQR* interquartile range, *SEER* surveillance, epidemiology, and end results

**Fig. 1:**
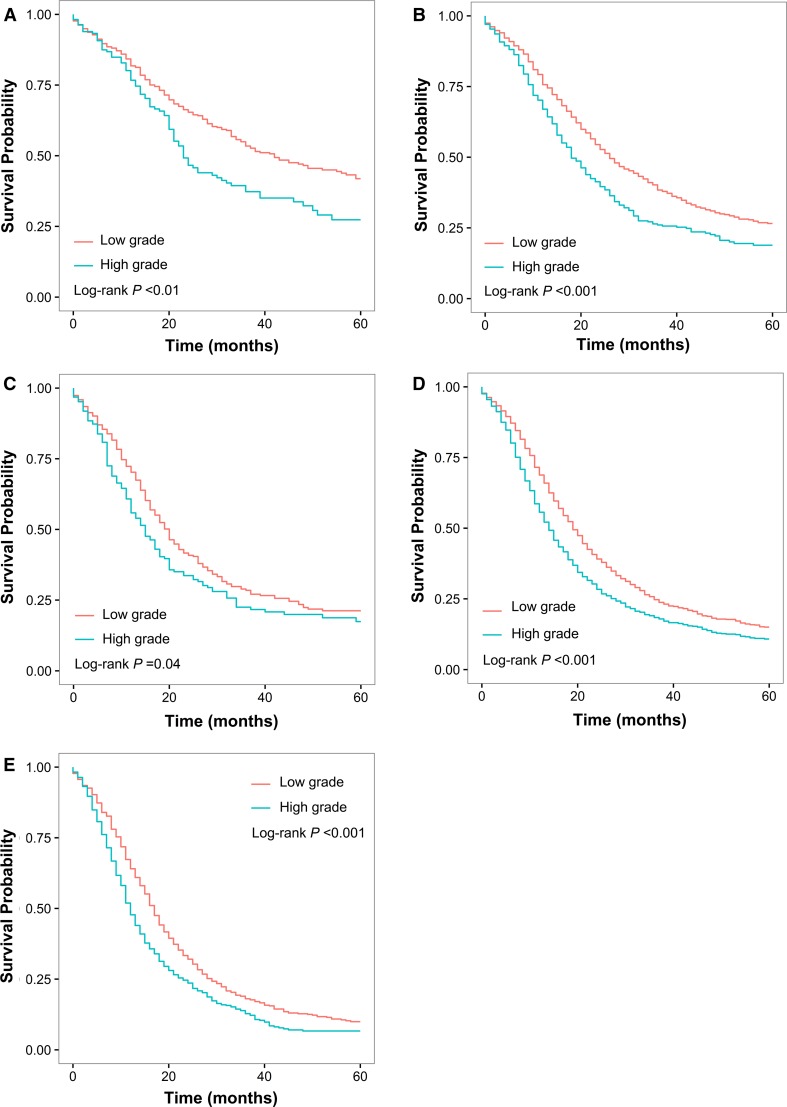
Overall survival for the study cohort of 7719 patients with resectable pancreatic ductal adenocarcinoma. Overall survival of patients with 8th AJCC **a** IA, **b** IB, **c** IIA, **d** IIB, and **e** III disease when stratified by tumor grade. Significant prognostic heterogeneity was identified in all 8th AJCC stages

After adjusted for race, year of diagnosis, age, sex, marital status, SEER region, tumor site, 8th T and N classification, and examined node count, high tumor grade was significantly associated with increased risk of death (HR 1.37; 95 % CI 1.29–1.44; *P* < 0.001). For each 8th AJCC stage, survival was significantly worse with high-grade versus low-grade disease (*P* < 0.01, *P* < 0.001, *P* = 0.04, *P* < 0.001, and *P* < 0.001 in 8th stage IA, IB, IIA, IIB, and III, respectively; Fig. 1a–e). Of note, patients with 8th stage IA disease (median survival: 36 months) were further stratified into subgroups with remarkably different OS, and an almost 20-month difference of median survival was identified between patients with low-grade tumor and those with high-grade tumor (42 vs. 23 months, *P* < 0.01; Fig. 1a).

**Fig. 2:**
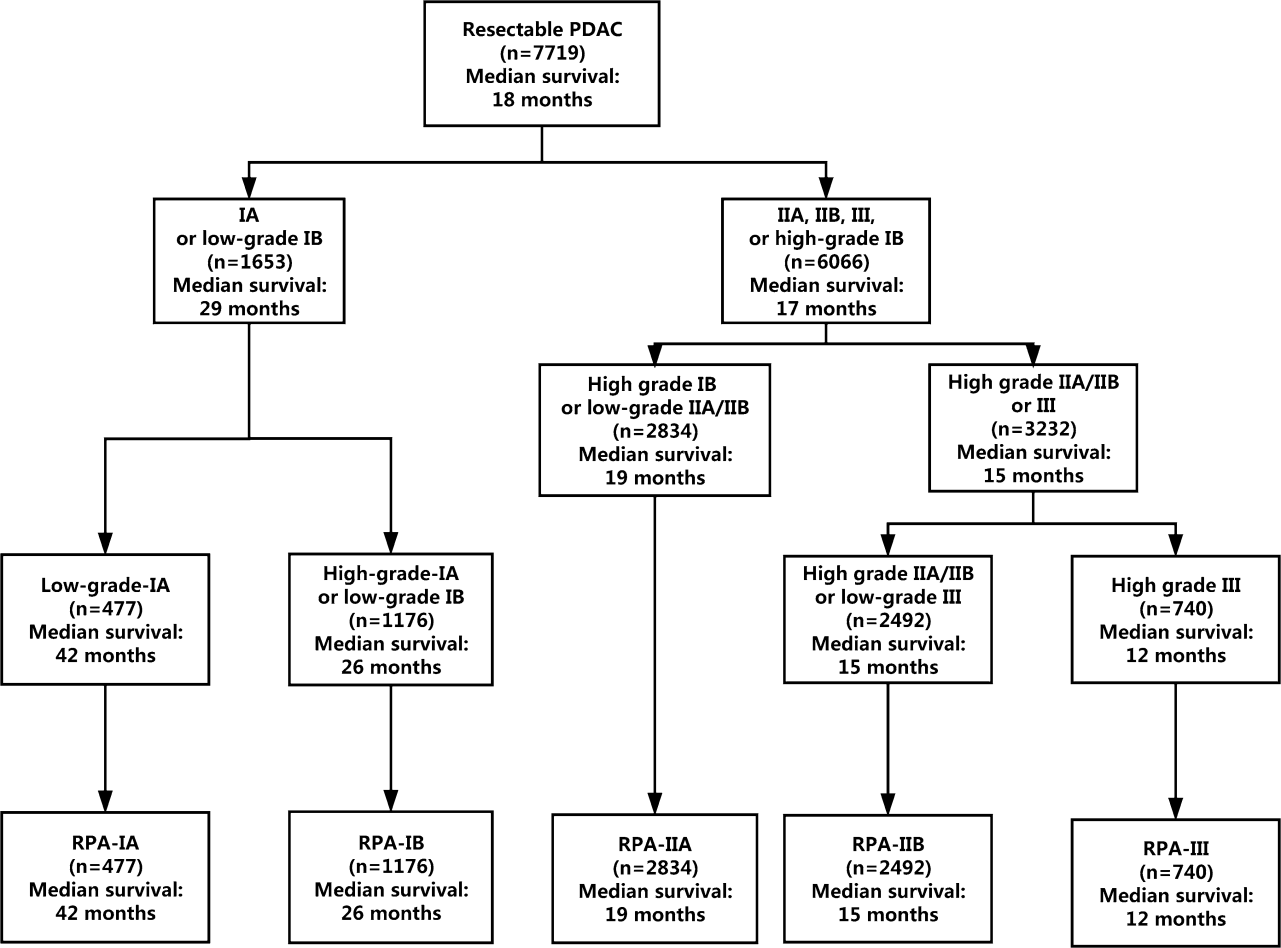
Refined stage grouping for resectable pancreatic ductal adenocarcinoma on the basis of RPA

**Fig. 3:**
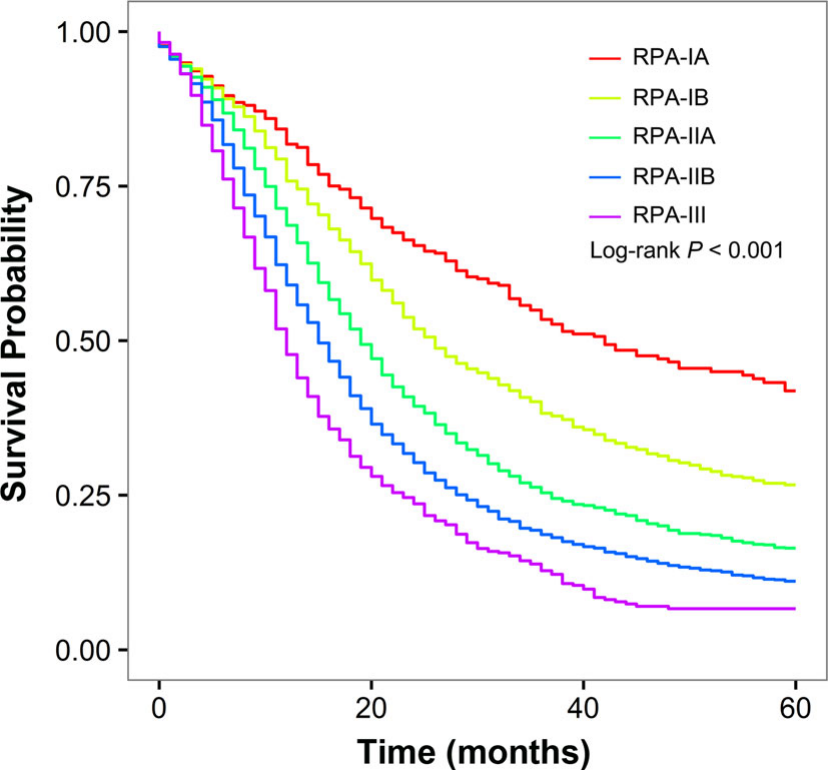
Overall survival of patients with resectable pancreatic ductal adenocarcinoma stratified by RPA stage. Each RPA stage represents a distinct prognosis

The RPA algorithm classified patients with resectable PDAC into the following five stage groups (Fig. 2): RPA-IA (low-grade T1N0), RPA-IB (high-grade T1N0 or low-grade T2N0), RPA-IIA (high-grade T2N0, low-grade T3N0, or low-grade T1–T3N1), RPA-IIB (high-grade T3N0, high-grade T1–T3N1, or low-grade T1–T3N2), and RPA-III (high-grade T1–T3N2). The RPA-IA, RPA-IB, RPA-IIA, RPA-IIB, and RPA-III stage groups included 477 (6.2 %), 1176 (15.2 %), 2834 (36.7 %), 2492 (32.3 %), and 740 (9.6 %) patients, respectively. The corresponding median survival was 42, 26, 19, 15, and 12 months, respectively (*P* < 0.001; Fig. 3). After adjustment for race, year of diagnosis, age, sex, marital status, SEER region, tumor site, tumor grade, and examined node count, we confirmed that a higher RPA stage was associated with an increased risk of death (RPA-IB vs. RPA-IA: HR, 1.49; RPA-IIA vs. RPA-IA: HR, 2.15; RPA-IIB vs. RPA-IA: HR 2.86; RPA-III vs. RPA-IA: HR, 3.96; *P* < 0.001 for all).

The RPA staging achieved a C-index of 0.585 (95 % CI 0.576–0.594), which was superior to the 8th AJCC staging scheme (C-index, 0.565; 95 % CI 0.556–0.573; *P* < 0.001). The bootstrap-corrected C-index for the RPA staging maintained to be 0.585, indicating minimal evidence of model overfit.

**Fig. 4:**
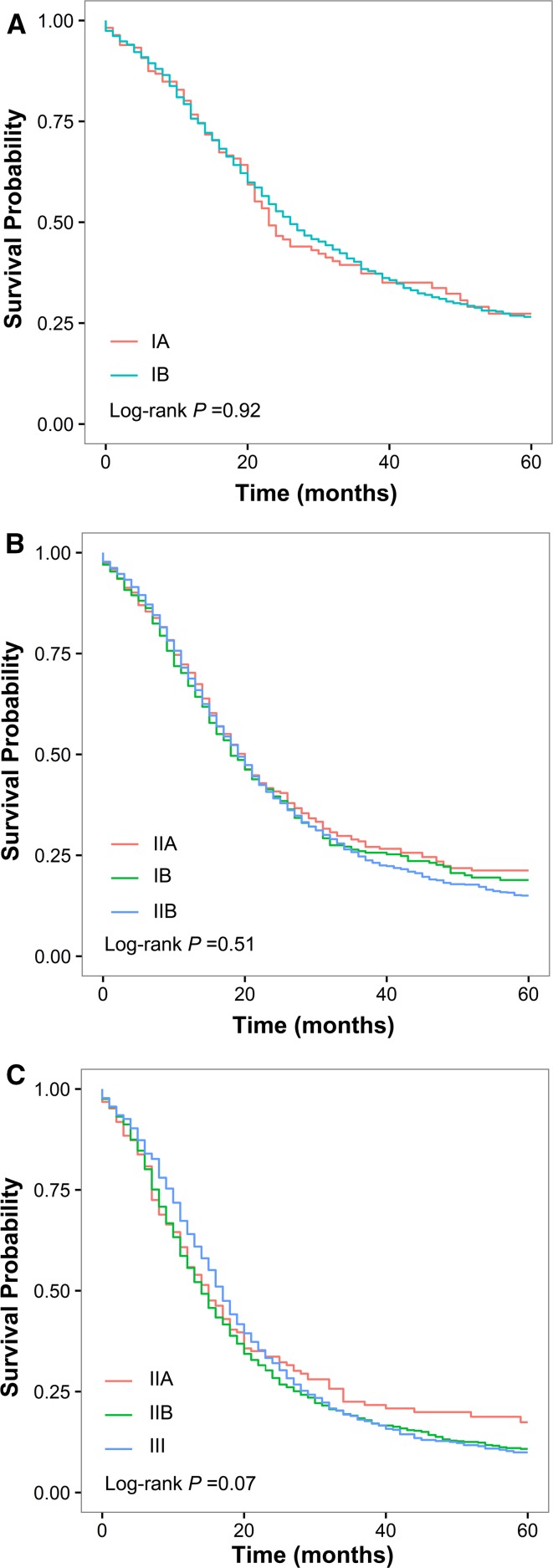
Assessment of the prognostic homogeneity of the RPA staging when assessed against the 8th AJCC staging. Overall survival of patients with **a** RPA-IB, **b** RPA-IIA, and **c** RPA-IIB disease when stratified by 8th AJCC stage

As shown in Table 2 and Fig. 4a–c, the RPA staging exhibited excellent prognostic homogeneity when assessed against the 8th AJCC staging; that is, for patients within each RPA stage, survival was homogeneous when stratified by 8th AJCC stages. Of note, patients with high-grade T1N0 and low-grade T2N0 tumors, who were classified into 8th stage IA and IB, respectively (median survival, 36 and 24 months, respectively), actually have similar survival (median survival, 23 and 26 months, respectively; *P* = 0.92) and were both classified into RPA-IB (Fig. 4a). In contrast, for patients within each 8th AJCC stage, the RPA staging can further stratify the patients into subgroups with remarkably different OS (*P* < 0.01, *P* < 0.001, *P* = 0.04, *P* < 0.001, and *P* < 0.001 in 8th stage IA, IB, IIA, IIB, and III, respectively; Table 2).

**Table 2:** Comparison of prognostic homogeneity between the 8th AJCC staging and the RPA staging

Staging scheme	RPA-IA		RPA-IB		RPA-IIA		RPA-IIB		RPA-III		*P* value^*^
	No.	Median survival	No.	Median survival	No.	Median survival	No.	Median survival	No.	Median survival	
*8th AJCC stage*											
IA	477	42 months	166	23 months	–	–	–	–	–	–	**<0.01**
IB	–	–	1010	26 months	470	18 months	–	–	–	–	**<0.001**
IIA	–	–	–	–	340	20 months	187	15 months	–	–	**0.04**
IIB	–	–	–	–	2024	19 months	1293	14 months	–	–	**<0.001**
III	–	–	–	–	–	–	1012	17 months	740	12 months	**<0.001**
*P* value^§^		–		0.92		0.51		0.07		–	

*AJCC* American Joint Committee on Cancer, *RPA* recursive partition analysis

* Comparison of median survival within different RPA stages. Bold *P* values indicate statistical significance (i.e., *P* < 0.05)

^§^ Comparison of median survival within different 8th AJCC stages. Bold *P* values indicate statistical significance (i.e., *P* < 0.05)

## Discussion

Grade is a measure of the degree of tumor differentiation. For PDAC, histologic grade is based on the extent of glandular differentiation. Several previous studies have detected the association between high tumor grade and adverse prognosis in patients with resected PDAC [4–8]. In this study of patients with resectable PDAC from the SEER database, we demonstrated significant prognostic heterogeneity within each 8th AJCC stage stratified by tumor grade, which verifies the prognostic value of tumor grade and suggests a room for improvement in the 8th AJCC staging scheme. Thus, we performed RPA to derive a new staging scheme of resectable PDAC which incorporated the prognostic impact of tumor grade and 8th AJCC stage.

The RPA staging scheme outperformed the 8th AJCC staging scheme in terms of discriminatory power and prognostic homogeneity. Although the discrimination was only moderately better, the prognostic homogeneity was considerably better for the RPA staging system. On the one hand, OS was homogeneous within each RPA stage regardless of the 8th AJCC stages. For example, high-grade T1N0 and low-grade T2N0 tumors, which were classified into different prognostic subgroups on the basis of the 8th AJCC staging (8th stage IA and IB, respectively), actually had similar survival and both were re-classified into RPA-IB. On the other hand, each 8th AJCC stage group could be classified by the RPA system into subgroups with remarkably different OS rates. For example, the 8th stage IA disease (8th T1N0) was further stratified into RPA-IB and RPA-IA disease depending on tumor grade, and the difference in median survival between patients in these two groups was almost 20 months.

Wasif et al. [7] have combined tumor grade and the 7th AJCC stage into a novel TNMG system, which exhibited superior survival discrimination to the 7th AJCC staging scheme. However, this scheme was based on arbitrary regrouping of patients within different risk groups, which is methodologically not sound [7]. Moreover, the TNMG system was derived from the 7th AJCC staging, of which the T3 classification (resectable PDAC extending beyond the pancreas) exhibited poor reproducibility among pathologists [14]. In contrast, the current RPA staging scheme was built upon the objective combination of tumor grade and the newly proposed 8th AJCC stage, of which the T classification has shown favorable reproducibility among different institutions [9].

For more accurate survival prediction in patients with PDAC, extensive efforts have been made for the development of prognostic nomograms which combined various prognosticators, such as the one created by the Memorial Sloan-Kettering Cancer Center [4]. However, these nomograms have not been widely accepted by patients and clinicians, probably because they are cumbersome to apply and inherently complex. In contrast, although the proposed RPA staging scheme was built upon the combination of tumor grade and 8th AJCC stage, it is a simple system which consists of five well-defined stage groups. Thus, it is of importance that the improved prognostic performance of the RPA staging scheme over the 8th AJCC staging scheme was not at the cost of complexity and ease of use in prognosis and treatment planning.

The proposed RPA staging scheme is clinically meaningful under the current treatment modality of resectable PDAC. Currently, international guidelines recommend adjuvant chemotherapy followed by curative surgery of PDAC, with the optimal chemotherapy regimen remaining unsettled [15, 16]. Additionally, the OS benefit from adjuvant chemotherapy was modest (difference in median OS: <5 months) according to the results of the CONKO-001 trial [17] and the ESPAC-1 trial [18]. The proposed RPA staging scheme, which had superior prognostic performance to the 8th AJCC staging, will be clinically useful in treatment planning, such as evaluating the administration of adjuvant chemotherapy and the trade-offs between chemotherapy regimens. Moreover, it may also help risk stratification of patients entering future clinical trials.

The present study has several limitations. First, even though the SEER database is checked regularly for discrepancy and reportedly has 95 % accuracy, the possibility of coding errors remains. Additionally, the measurement of tumor size of PDAC may not always be accurate due to the difference in the percentage of tumor mesenchyme, the condition of chronic pancreatitis, and the experience of pathologists. Moreover, because information regarding adjuvant chemotherapy was not available in the SEER database, we were not able to assess how the proposed RPA staging may influence patient selection for adjuvant chemotherapy. Finally, although the proposed RPA staging performed well during internal validation using bootstrapped resampling, external validation using patient cohorts from other countries outside the USA is required. Despite these limitations, the use of the SEER data enables us to draw solid conclusions pertinent to the general clinical practice on the basis of a large sample of patients with PDAC, which is not possible in single-institution studies.

In summary, we demonstrated that high tumor grade was associated with poor prognosis among patients with resectable PDAC across all 8th AJCC stages, suggesting the 8th AJCC staging needed improvement. Accordingly, we used RPA to develop a refined staging scheme which incorporated the prognostic information of tumor grade and 8th AJCC stage for patients with resectable PDAC. The RPA staging outperformed the 8th AJCC staging but was not substantially more complex. This newly proposed staging system will be clinically useful for prognosis, surveillance, and treatment planning, as well as risk stratification in future clinical trials for patients with resectable PDAC.
